# Complete mitochondrial genome of black-shanked douc langurs (*Pygathrix nigripes*) and its phylogenetic analysis

**DOI:** 10.1080/23802359.2018.1512386

**Published:** 2018-10-08

**Authors:** Qian Su, Chongtao Chen, Dingju Wei, Diyan Li, Huaming Xu, Jiayun Wu, Anxiang Wen, Meng Xie, Qin Wang, Guangxiang Zhu, Qingyong Ni, Mingwang Zhang, Huailiang Xu, Yongfang Yao

**Affiliations:** a College of Life Science, Sichuan Agricultural University, Ya’an, China;; b Nanning Zoo, Nanning, China;; c College of Animal Science and Technology, Sichuan Agricultural University, Chengdu, China

**Keywords:** Cercopithecoidea, *Pygathrix nigripes*, mitochondrial genome, phylogenetic analysis

## Abstract

In this study, we first characterized the complete mitogenome of *Pygathrix nigripes*, and analysed its phylogenetic status. The circular mitogenome was 16,534 bp in length, and contained 13 protein-coding genes (PCGs), two rRNA genes, 22 tRNA genes and one non-coding control region (D-loop). These genes except ND6 and 8 tRNA genes were encoded on the H-strand. The phylogenetic analysis exhibited that our sequence formed a sister branch with *P. cinereal* and *P. nemaeus* of genus *Pygathrix*, which showed a closer genetic relationship of the three species. These information contribute to molecular, phylogenetic studies and genetic diversity conservation for this species.

Black-shanked douc langur (*Pygathrix nigripes*), taxonomically affiliated to the subfamily Colobinae in the family Cercopithecidae (Hasegawa et al. 2002; Duc et al. 2009), mainly inhabited evergreen/semi-evergreen forests and rainforests in eastern Cambodia and southeast Vietnam with a highly narrow habitat range (Thuc et al. 2005; Bett et al. 2012). Owing to illegal hunting for traditional ‘medicine’, pet trade and natural habitat destruction, the wild populations of *P. nigripes* have reduced dramatically (Nadler 2008), and it was classified as Endangered (EN) in the International Union for Conservation of Nature (IUCN 2007) Red List of Threatened Species (Rawson et al. 2008). In the present paper, we first determined and characterized the complete mitogenome of *P. nigripes* to contribute to its molecular, phylogenetic studies and genetic diversity conservation.

The muscle tissue of the black-shanked douc langur was collected from a natural death individual in Nanning, China (22°50′N, 108°15′E), and it was returned to the zoology lab and stored at −80 °C in Sichuan Agricultural University. Genomic DNA was extracted by phenol-chloroform extraction method (Sambrook et al. 1990). Sequences’ homology was analyzed using Megalign of the DNAStar (Swayne et al. 2015). Nucleotide variation loci were calculated by DnaSP v5 (Librado and Rozas 2009). Phylogenetic tree was constructed using Mega 7.0 (Kumar et al. 2016).

The complete mitogenome sequence of *P. nigripes* (GenBank accession number MH064177) was 16,534 bp, including 13 PCGs, two rRNA genes (*12S rRNA* and *16S rRNA*), 22 tRNA genes and one control region (D-loop). Genes encoding on the genome were similar among all primates (Roos 2018; Roos et al. 2018). The overall base composition is 32.4% A, 29% T, 25.9% C, 12.7% G, and the G + C content was 38.6%. Most PCGs started with ATG, while *NADH2* and *NADH3* genes initiated with ATT, *NADH5* began with ATA, and *NADH6* used TCT as start codon. All PCGs were terminated with typical TAA or TAG codons except for *COX3, NADH3, NADH4*, *CYTB,* which ended with incomplete stop codon AT- or T–, and *NADH6* ended with CAT. These genes except ND6 and eight tRNA genes were encoded on the H-strand. The lengths of *12S rRNA* and *16S rRNA* were 1020 bp and 1086 bp, and separated by the *tRNA^Val^* gene. The D-loop was 1092 bp in length, and located between *tRNA^Pro^* and *tRNA^Phe^*.

Phylogenetic analysis included mitogenome of *P. nigripes* and the other 12 species that are from Cercopithecinae and Colobinae, which belong to Primates, using *Callithrix jacchus* (Callithrix) as an outgroup (Figure 1). The neighbour-joining (NJ) analysis exhibited that our sequence formed a sister branch with *P. cinereal* and *P. nemaeus* of genus *Pygathrix*, which showed a closer genetic relationship of the three species. Furthermore, the homology of 13PCGs between *P. nigrip*es with *P. nemaeus*, *P. cinereal* is 92.75%, 92.51%, respectively, while 98.97% between *P. cinereal* and *P. nemaeus*, it suggested that *P. cinereal* and *P. nemaeus* have a closer genetic relationship. The result is in line with the topology of phylogenetic tree. This study provides new and comprehensive insight into the evolutionary and biogeographic history of this species, and contributes additional molecular information to species conservation.

**Figure 1.**
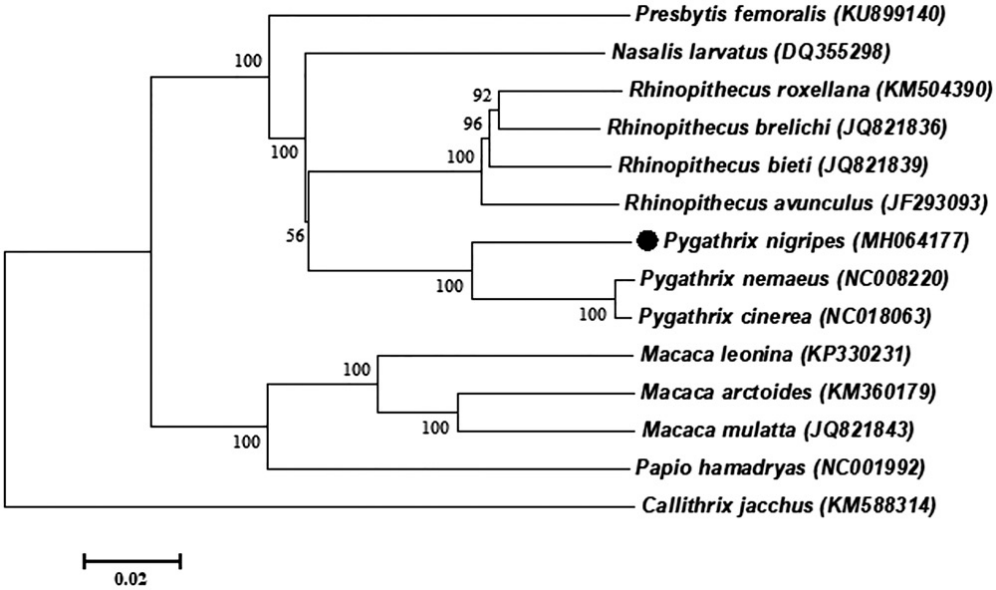
NJ phylogenetic tree based on the complete mitochondrial genome of the *P. nigripes* and other 12 primates species sequences. *Callithrix jacchus* was served as an outgroup. Numbers at the branches indicated the bootstrapping values with 1000 replications. GenBank accession numbers were given in the parentheses. Filled circle represented a sequence from this study.
